# Modulation of AMPK/ TET2/ 5-hmC axis in response to metabolic alterations as a novel pathway for obesity-related colorectal cancer development

**DOI:** 10.1038/s41598-023-29958-2

**Published:** 2023-02-17

**Authors:** Takashi Kon, Yu Sasaki, Yasuhiko Abe, Yusuke Onozato, Makoto Yagi, Naoko Mizumoto, Takayuki Sakai, Matsuki Umehara, Minami Ito, Shuhei Nakamura, Hiroki Goto, Yoshiyuki Ueno

**Affiliations:** 1 Faculty of Medicine, Department of Gastroenterology, Faculty of Medicine, Yamagata University, 2-2-2 Iida-Nishi, Yamagata, 990-9585 Japan; 2 Division of Endoscopy, Yamagata University Hospital, 2-2-2 Iida-Nishi, Yamagata, 990-9585 Japan

**Keywords:** Colorectal cancer, Obesity

## Abstract

Obesity is a major risk factor for colorectal cancer (CRC). Sustained hyperglycemia destabilizes tumor suppressor ten-eleven translocation (TET) 2, which is a substrate of AMPK, thereby dysregulating 5-hydroxymethylcytosine (5-hmC). However, the role played by this novel pathway in the development of obesity-related CRC is unclear. In this study, we aimed to evaluate the expression levels of TET2 and 5-hmC in obesity-related CRC and the effects of TET2 expression on the proliferation of CRC cells. To this end, surgically resected CRC samples from seven obese patients (Ob-CRC) and seven non-obese patients (nOb-CRC) were analyzed, and expression levels of the TET family and 5-hmC were compared between the groups. A decrease was observed in *TET2* mRNA levels and 5-hmC levels in Ob-CRC compared to that in nOb-CRC. Furthermore, we used CRC cell lines to investigate the relationship between insulin, proliferation, and *TET* expression and AMPK. In cell lines, glucose and insulin treatments suppressed the expression of *TET2* and increased cell proliferation. Downregulation of *TET2* using siRNA also induced cell proliferation. An AMPK activator inhibited insulin- or glucose-stimulated cell proliferation and restored TET2 expression. We propose the AMPK-TET2-5-hmC axis as a novel pathway and potential therapeutic target in obesity-related CRC development.

## Introduction

Colorectal cancer (CRC) is the third most prevalent cancer and the second leading cause of mortality worldwide. In 2018, it was estimated that CRC caused approximately 881,000 deaths globally^1^. In Japan, there is an increase in the mortality and morbidity rates associated with CRC. The increase in obesity and metabolic syndrome caused by the adoption of a western-style diet and physical inactivity is a contributing factor^2^. Insulin resistance and alterations in levels of adipocytokines, along with visceral fat obesity, play a significant role. Hyperinsulinemia after insulin resistance in visceral fat obesity, abnormal secretion of adipocytokines^3–5^, chronic low-grade inflammation^6,7^, and altered secretion of incretin^8,9^ are important factors in the development of obesity-related colorectal carcinogenesis and its precancerous lesion, colorectal adenoma (CRA). However, the direct molecular mechanisms by which obesity and metabolic syndrome lead to CRC development remain to be elucidated.

Genetic alterations in cancer genes and epigenetic alterations through base modifications without sequence changes are important factors in carcinogenesis^10^. One of the epigenetic changes associated with carcinogenesis is aberrant DNA methylation. DNA derived from cancerous tissues exhibits two kinds of changes in the amount of methylated cytosine: a reduction throughout the genome and an increase in certain promoter regions (CpG islands). Both changes contribute to carcinogenesis^11^. Excessive methylation of the promoter regions of tumor suppressor genes inactivates these genes^12^, while genome-wide DNA hypomethylation induces chromosomal instability, leading to carcinogenesis. A subset of colorectal cancers is associated with the accumulation of CpG island hypermethylation in specific promoter regions and, therefore, is referred to as the CpG island methylator phenotype (CIMP)^13^. In CIMP-positive CRCs, the epigenetic silencing of mismatch repair genes, such as *MLH1* (*MutL homolog 1*), is frequently observed with a high degree of methylation^14^. Moreover, CIMP may play a role in a colorectal carcinogenic pathway called the serrated neoplastic pathway^15^. The serrated neoplastic pathway as well as the classical adenoma-carcinoma sequence are associated with a precancerous lesion called traditional serrated adenoma^16^. Hypermethylation of the secreted protein acidic and rich in cysteine–related modular calcium-binding protein 1 gene is involved in the development of this lesion.^16^ Therefore, aberrant DNA methylation possibly plays an important role in multiple molecular pathways that lead to colorectal carcinogenesis.

The ten-eleven translocation (TET) protein catalyzes the base conversion of 5-methylcytosine (5-mC) to 5-hydroxymethylcytosine (5-hmC), which is a key intermediate product in the DNA demethylation reaction^17^. TET contains three subtypes—TET1, TET2, and TET3. *TET2* mutations are frequently observed in hematological malignancies, such as myelodysplastic syndromes and acute myeloid leukemia^18^. Additionally, *TET2* mutations are associated with the development of solid tumors, including breast cancer tumors^19^. The expression levels of *TET1* and *TET2* are critical in CRC—*TET1* is downregulated in cancer tissues, and this suppression of *TET1* inhibits the proliferation of CRC cell lines^20^. In addition, decreased expression of *TET2* is an independent poor prognostic factor for patients with CRC^21^. Therefore, abnormalities in the TET family related demethylation may influence the development of CRC.

Hyperglycemia reduces the activity of a cellular energy sensor called adenosine monophosphate-activated protein kinase (AMPK), induces instability of TET2, and promotes carcinogenesis^22^. A novel molecular mechanism has been unveiled in which systemic metabolic alterations directly affect epigenetics, leading to carcinogenesis. A functional knockdown of *TET2* in hematopoietic cells increases the expression of interleukin-1β (IL-1β) in the white adipose tissue and induces systemic insulin resistance^23^. Glucose intolerance and insulin resistance are critical features of obesity and metabolic syndrome, and they are significant factors in the development and progression of obesity-related CRC. These findings suggest that TET2 plays an important role in colorectal carcinogenesis associated with obesity and metabolic syndrome; however, the mechanism of action remains to be investigated. In this study, we evaluated the expression levels of TET2 and 5-hmC in obesity-related CRC and the effects of TET2 expression on the proliferative activity in CRC cells.

## Results

### Elevated plasma glucose and insulin levels and insulin resistance are observed in obese patients with CRC

The characteristics of the 14 enrolled subjects are summarized in Supplementary Table 1. There were no significant differences in median age, sex, current smoking habits, and alcohol consumption between the obese patients with CRC (Ob-CRC) and the non-obese patients with CRC (nOb-CRC). The median visceral fat area (VFA, *p* < 0.01) and body mass index (BMI, *p* < 0.01) were significantly higher in the Ob-CRC group than in the nOb-CRC group. Compared to the nOb-CRC group, the Ob-CRC group had significantly higher median levels of fasting plasma glucose (FPG, *p* < 0.01), fasting plasma insulin (FPI, *p* < 0.01) and the homeostasis model assessment of insulin resistance (HOMA-IR) values (*p* < 0.01). No significant differences were observed between the Ob-CRC and nOb-CRC groups in terms of diabetes, dyslipidemia, and stages, and the median levels of hemoglobin A1c (HbA1c), triglyceride (TG), total cholesterol (TC), high-density lipoprotein (HDL), and low-density lipoprotein (LDL) concentrations.

### 5-hmC levels are reduced in the colorectal cancer tissues from obese patients

**Figure 1:**
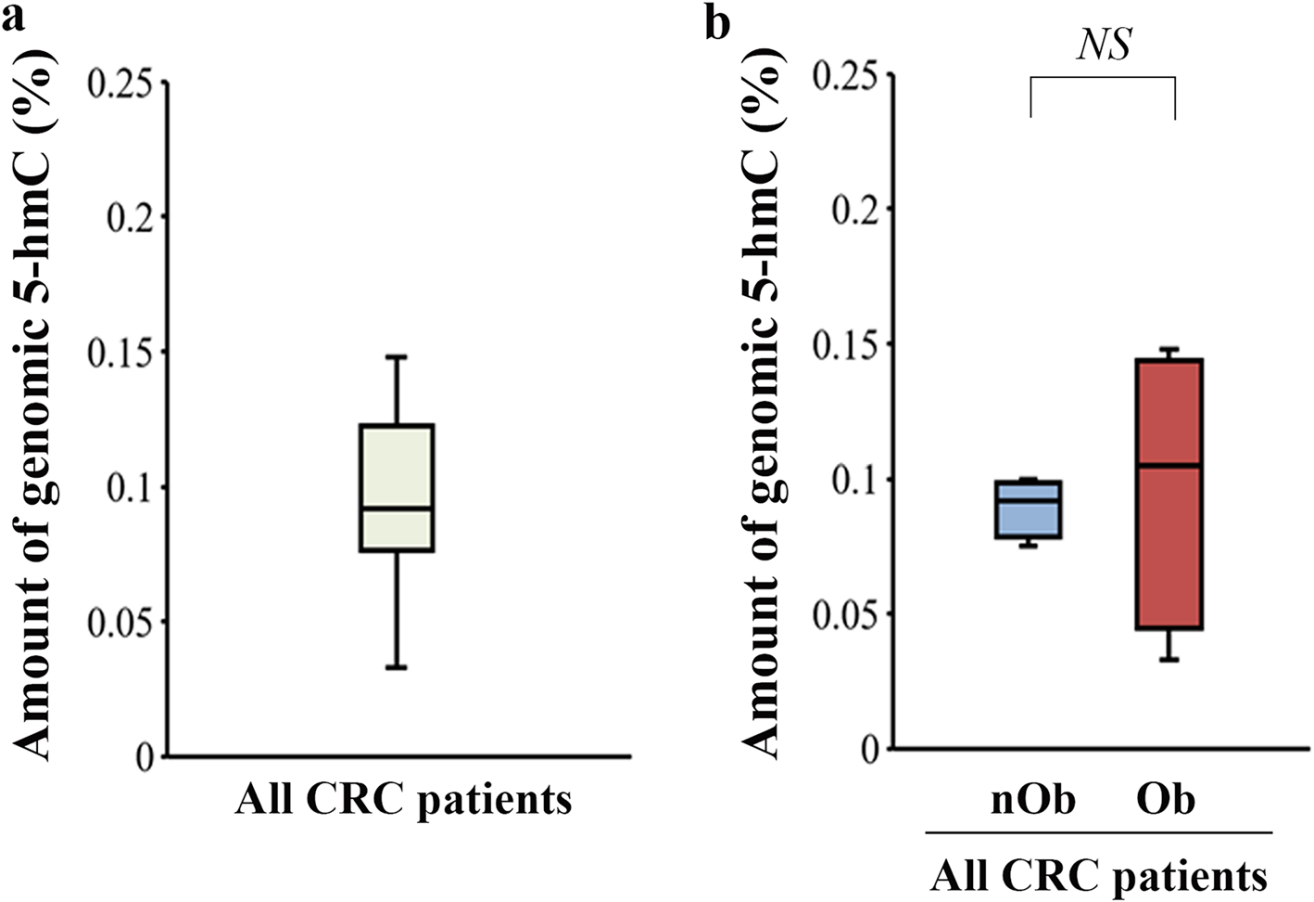
5-hmC levels of genomic DNA. In all CRC patients (**a**), the median percentage of 5-hmC from genomic DNA was 0.092% (IQR 0.076–0.123), which was not significantly different between nOb-CRC and Ob-CRC groups (*p* = 0.78, **b**). *NS* not significant, *IQR* interquartile range, *5-hmC* 5-hydroxymethylcytosine, *Ob-CRC* obese patients with colorectal cancer, *nOb-CRC* non-obese patients with colorectal cancer.

**Figure 2:**
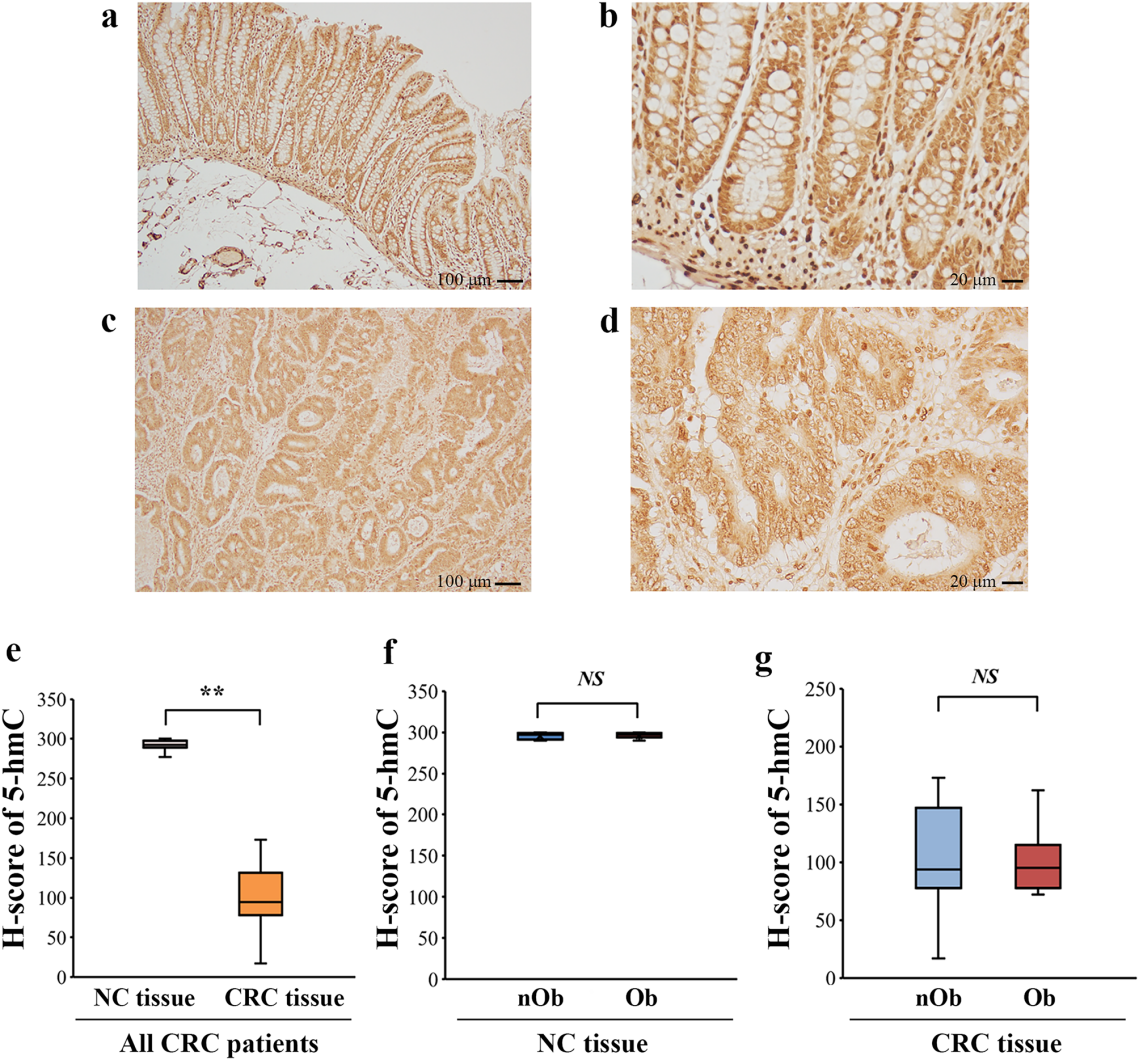
Analysis of immunohistochemical staining of 5-hmC. Strong nuclear signals of 5-hmC in the NC tissues (low-power field: **a**, high-power field: **b**) and decreased nuclear expression in CRC tissues (low-power field: **c**, high-power field: **d**). Quantitative analysis of nuclear staining intensity of 5-hmC with H-scores showed a decrease in the CRC tissues, compared to that in the NC tissues (*p* < 0.01, **e**); however, no difference was detected between Ob-CRC and nOb-CRC in both NC tissues (*p* = 0.95, **f**) and CRC tissues (*p* = 0.95, **g**). The H-scores for each case were calculated from the mean scores of three individual sections. **p < 0.01; *NS* no significant. *NC* adjacent noncancerous, *CRC* colorectal cancer, *5-hmC* 5-hydroxymethylcytosine, *Ob-CRC* obese patients with colorectal cancer, *nOb-CRC* non-obese patients with colorectal cancer.

**Figure 3:**
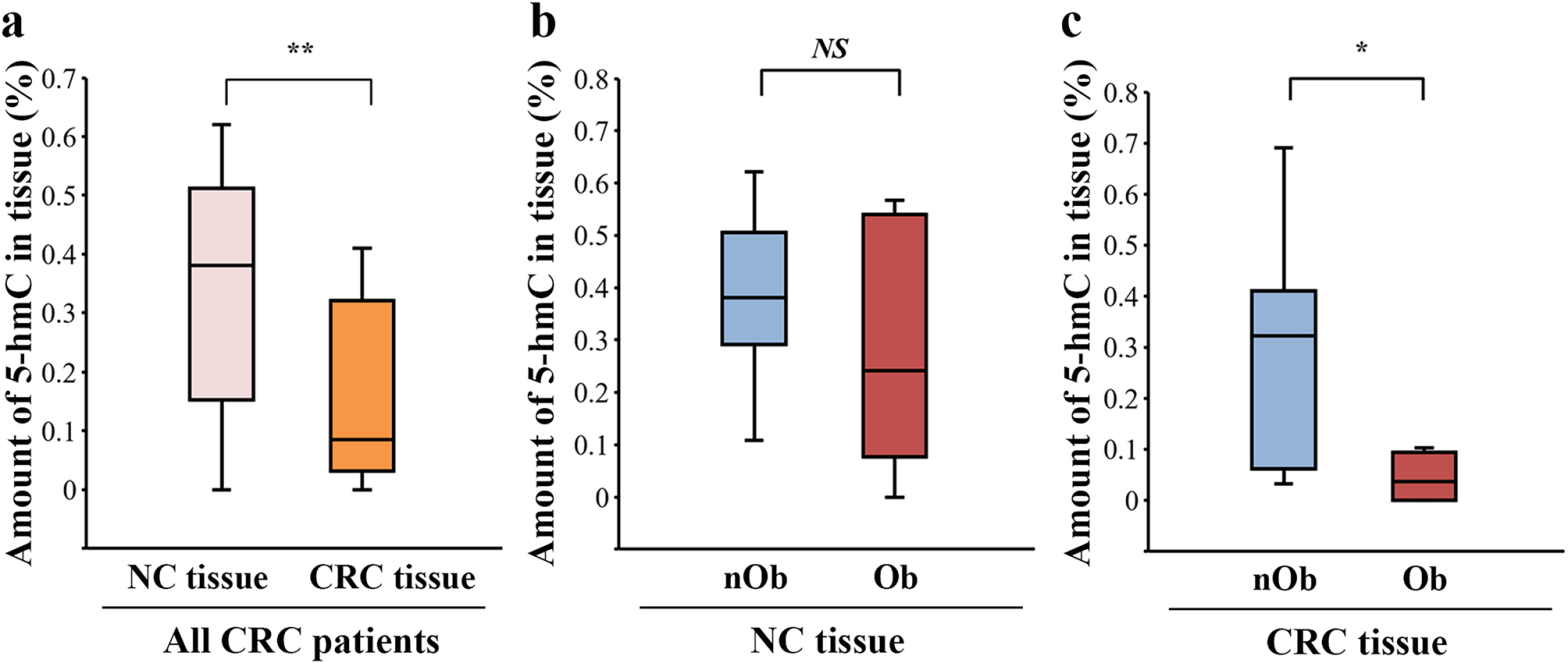
Quantitative determination of 5-hmC with ELISA using DNA isolated from FFPE tissues. Compared to the NC tissue, CRC tissue showed a decrease in 5-hmC as well as H-scores (*p* < 0.01, **a**). There was no difference in 5-hmC levels between the nOb-CRC and Ob-CRC groups in NC tissues (*p* = 0.46, **b**); however, there was a significant decrease in CRC tissues in the Ob-CRC group (*p* = 0.03, **c**). *p < 0.05; **p < 0.01; *NS*, no significant, *FFPE* formalin-fixed paraffin-embedded, *NC* the adjacent noncancerous, *CRC* colorectal cancer, *5-hmC* 5-hydroxymethylcytosine, *Ob-CRC* obese patients with colorectal cancer, *nOb-CRC* non-obese patients with colorectal cancer.

The median 5-hmC% of genomic DNA isolated from the blood of CRC patients was 0.092% (Fig. 1a), which was not significantly different between the nOb-CRC and Ob-CRC groups (*p* = 0.78, Fig. 1b). Immunohistochemistry indicated decreased nuclear expression of 5-hmC in CRC tissues, while strong nuclear signals were observed in the adjacent noncancerous (NC) tissues (Fig. 2a–d). The median H-score for nuclear staining intensity of 5-hmC in the CRC tissues was significantly lower than that in the NC tissues in all CRC patients (*p* < 0.01; Fig. 2e). No significant difference was detected in the median H-score for nuclear staining intensity of 5-hmC in either NC or CRC tissues, between the nOb-CRC and Ob-CRC groups (Fig. 2f, g). The percent of 5-hmC measured in the DNA isolated from formalin-fixed paraffin-embedded (FFPE) tissue (using ELISA), indicated that the median level of 5-hmC amount was significantly lower in CRC tissue, compared to that in NC tissue (*p* < 0.01; Fig. 3a). In the NC tissue, there was no significant difference between the nOb-CRC and Ob-CRC groups (*p* = 0.46; Fig. 3b). The median level of 5-hmC amount in CRC tissue was significantly lower in Ob-CRC, compared to that in nOb-CRC group (*p* = 0.03; Fig. 3c).

### *TET2* expression is reduced in obese patients with colorectal cancer

**Figure 4:**
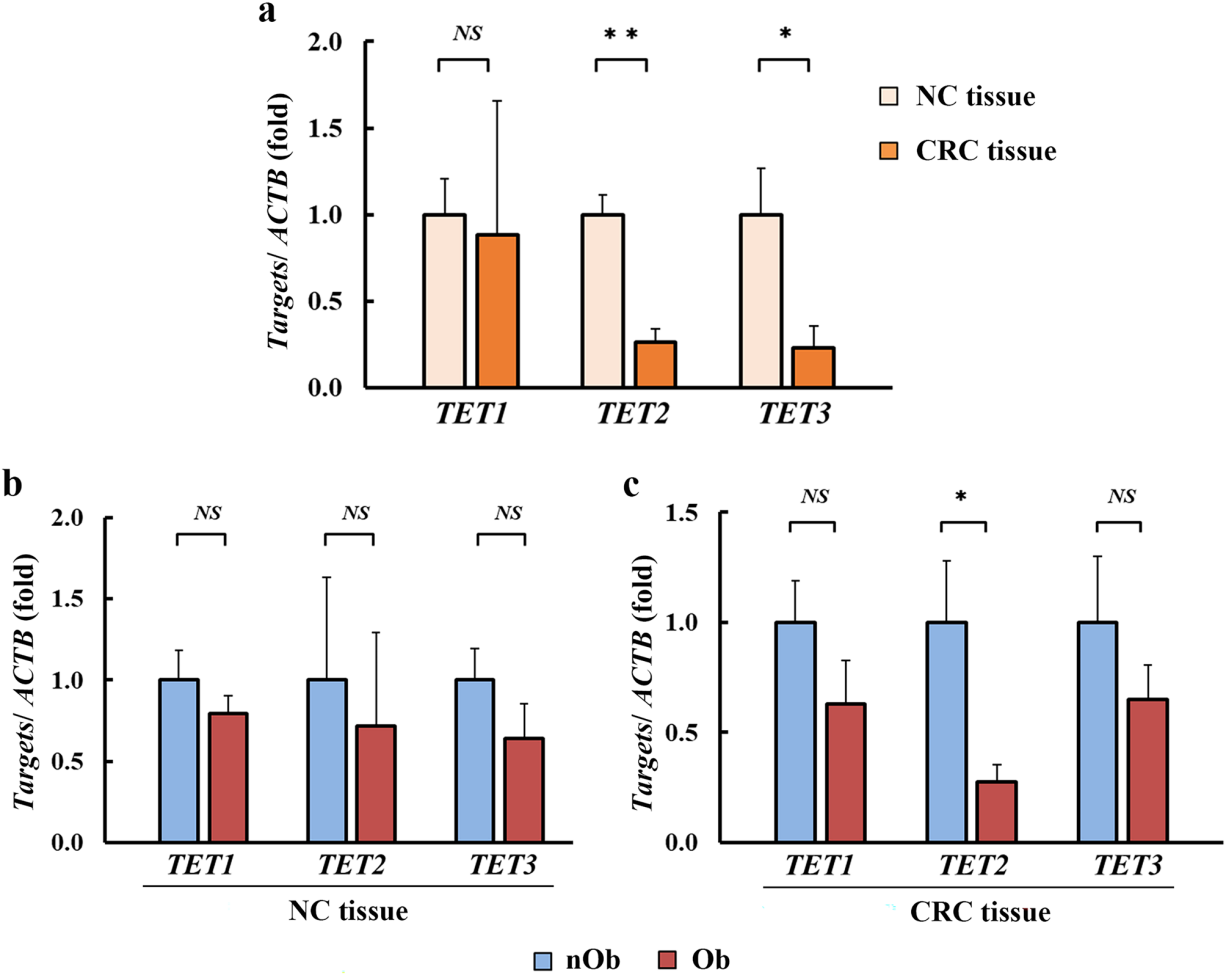
Expression analysis of *TET1* to *3* using RT-qPCR. Expression levels of *TET2* (0.26-fold,* p* < 0.01) and *TET3* (0.23-fold, *p* = 0.03) were significantly lower in CRC tissues, when compared to that in NC tissues (**a**), whereas no significant difference was observed in *TET1* expression (*p* = 0.89). In the NC tissue (**b**), no significant differences were detected in the expression levels of *TET1* (*p* = 0.72), *TET 2* (*p* = 0.21), and *TET 3* (*p* = 0.37). In CRC tissue (**c**), there was a lower expression of *TET2* in Ob-CRC, compared to that in the nOb-CRC group (0.27-fold, *p* = 0.04). *p < 0.05, **p < 0.01, *NS* no significant. *RT-qPCR* real-time quantitative polymerase chain reaction, *NC* adjacent noncancerous, *CRC* colorectal cancer, *TET* ten-eleven translocation, *ACTB* β-actin, *Ob-CRC* obese patients with colorectal cancer, *nOb-CRC* non-obese patients with colorectal cancer.

**Figure 5:**
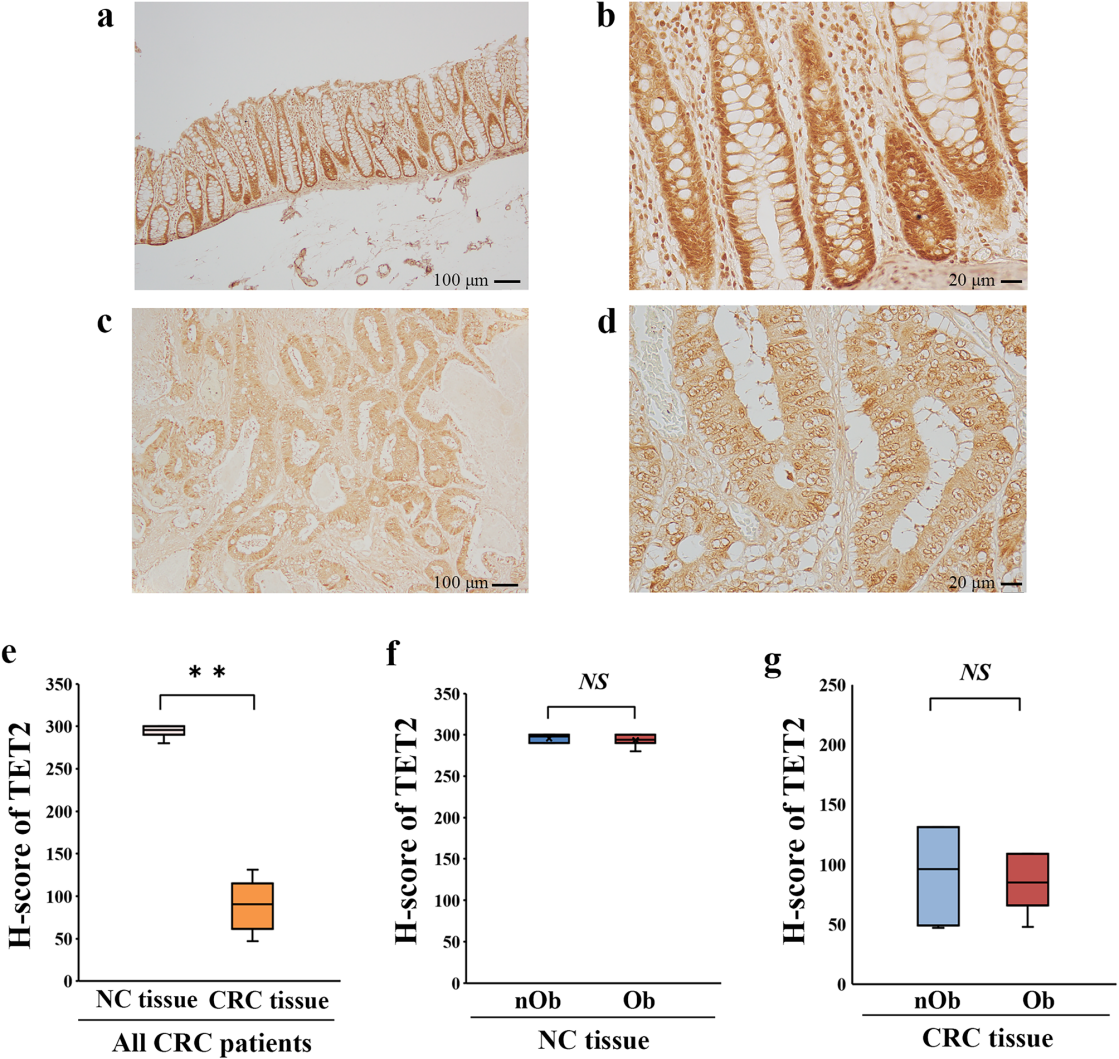
Analysis of immunohistochemical staining of TET2. Strong nuclear signals of TET2 in the NC tissues (low-power field: **a**, high-power field: **b**) and low expression of TET2 in the CRC tissues (low-power field: **c**, high-power field: **d**). Quantitative analysis of nuclear staining intensity of TET2 with H-scores indicated a decrease in the CRC tissue, when compared to that in the NC tissue (*p* < 0.01, **e**); however, no difference was detected between the Ob-CRC and nOb-CRC groups, with respect to both NC (*p* = 0.80, **f**) and CRC tissues (*p* = 0.80, **g**). The H-scores for each case were calculated from the mean scores of three individual sections. *p < 0.05; **p < 0.01; *NS* no significant. *NC* adjacent noncancerous, *CRC* colorectal cancer, *TET* ten-eleven translocation, *Ob-CRC* obese patients with colorectal cancer, *nOb-CRC* non-obese patients with colorectal cancer.

The expression levels of *TET1*, *TET2*, and *TET3* in NC and CRC were evaluated using quantitative reverse transcription-polymerase chain reaction (RT-qPCR). Compared to that in the NC tissue, the expression of *TET2* (*p* < 0.01) and *TET3* (*p* = 0.03) was significantly reduced in CRC tissue, whereas there was no significant difference in the expression of *TET1* (*p* = 0.89; Fig. 4a). No significant differences were detected in the expression levels of *TET1*,* 2*, and *3* in the NC tissues from both groups (Fig. 4b). In case of CRC tissues, significantly lower levels of *TET2* were observed only in Ob-CRC and not in the nOb-CRC group (*p* = 0.04, Fig. 4c). Immunohistochemistry revealed that the nuclear expression TET2, assessed using H-score, was significantly reduced in CRC tissues compared to that in the NC tissues (*p* < 0.01, Fig. 5a–e). There was no significant difference in the nuclear expression of TET2 in both NC and CRC tissues between the nOb-CRC and Ob-CRC groups (Fig. 5f, g).

### AMPK activation restores glucose- and insulin-induced TET2 downregulation in human colorectal cancer cells

**Figure 6:**
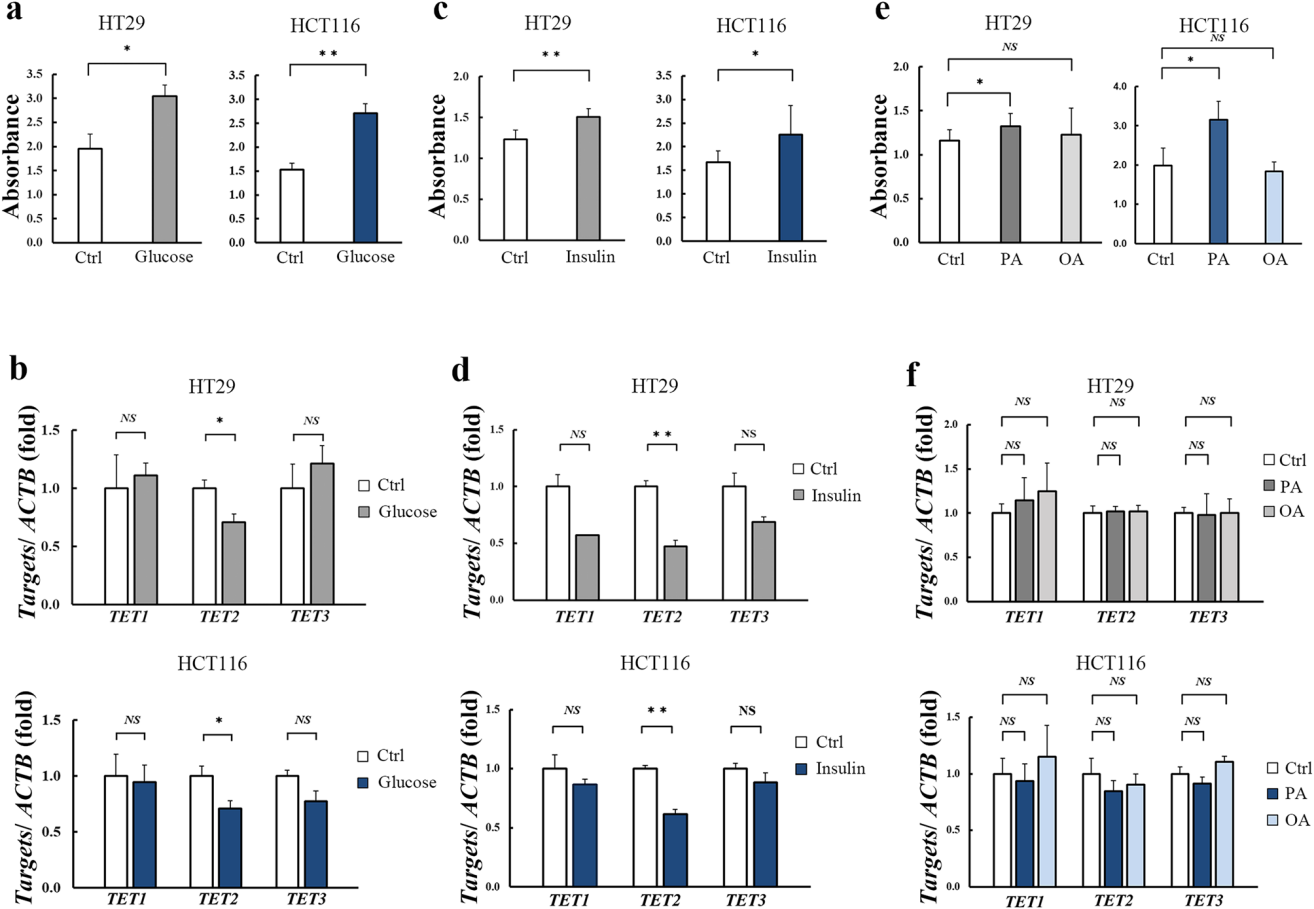
Effect of metabolic factors on the proliferation and *TET2* expression of human colorectal cancer cells. Treatment with 50 mM glucose-stimulated proliferation (**a**) and significantly decreased the expression of *TET2* mRNA (**b**) in both HT-29 (0.71-fold, *p* = 0.03) and HCT116 (0.71-fold, *p* = 0.04) cells. Similarly, treatment with 100 nM insulin-stimulated proliferation (**c**) and significantly decreased the expression of *TET2* mRNA (**d**) in both HT29 (0.47-fold, *p* < 0.01) and HCT116 (0.62-fold, *p* < 0.01). Treatment with 250 µM PA induced proliferation, while that with 250 µM OA did not induce any changes, in both cell lines (**e**), no significant alteration in the expression of *TET*s in either fatty acid treatments (**f**). *p < 0.05; **p < 0.01; *NS* no significant. *TET* ten-eleven translocation, *Ctrl* control, *PA* palmitic acid, *OA* oleic acid, *ACTB* β-actin.

To investigate the mechanism of downregulation of TET2 in Ob-CRC, human CRC cell lines HT29 and HT116 were treated with metabolic factors, such as glucose, insulin, a saturated fatty acid (palmitic acid [PA]), and an unsaturated fatty acid (oleic acid [OA]). Glucose treatment (Fig. 6a, b) stimulated cell proliferation and decreased the *TET2* expression in both HT-29 (0.71-fold, *p* = 0.03) and HCT116 (0.71-fold, *p* = 0.04) cells. Insulin treatment yielded similar results (0.47-fold, *p* < 0.01 for HT29; 0.62-fold, *p* < 0.01 for HCT116; Fig. 6c, d). PA treatment stimulated proliferation in both CRC cell lines; however, OA treatment had no significant effect on proliferation (Fig. 6e). There was no significant alteration in the expression of *TET1, 2,* and *3* in the CRC cell lines when treated with the fatty acids (Fig. 6f).

**Figure 7:**
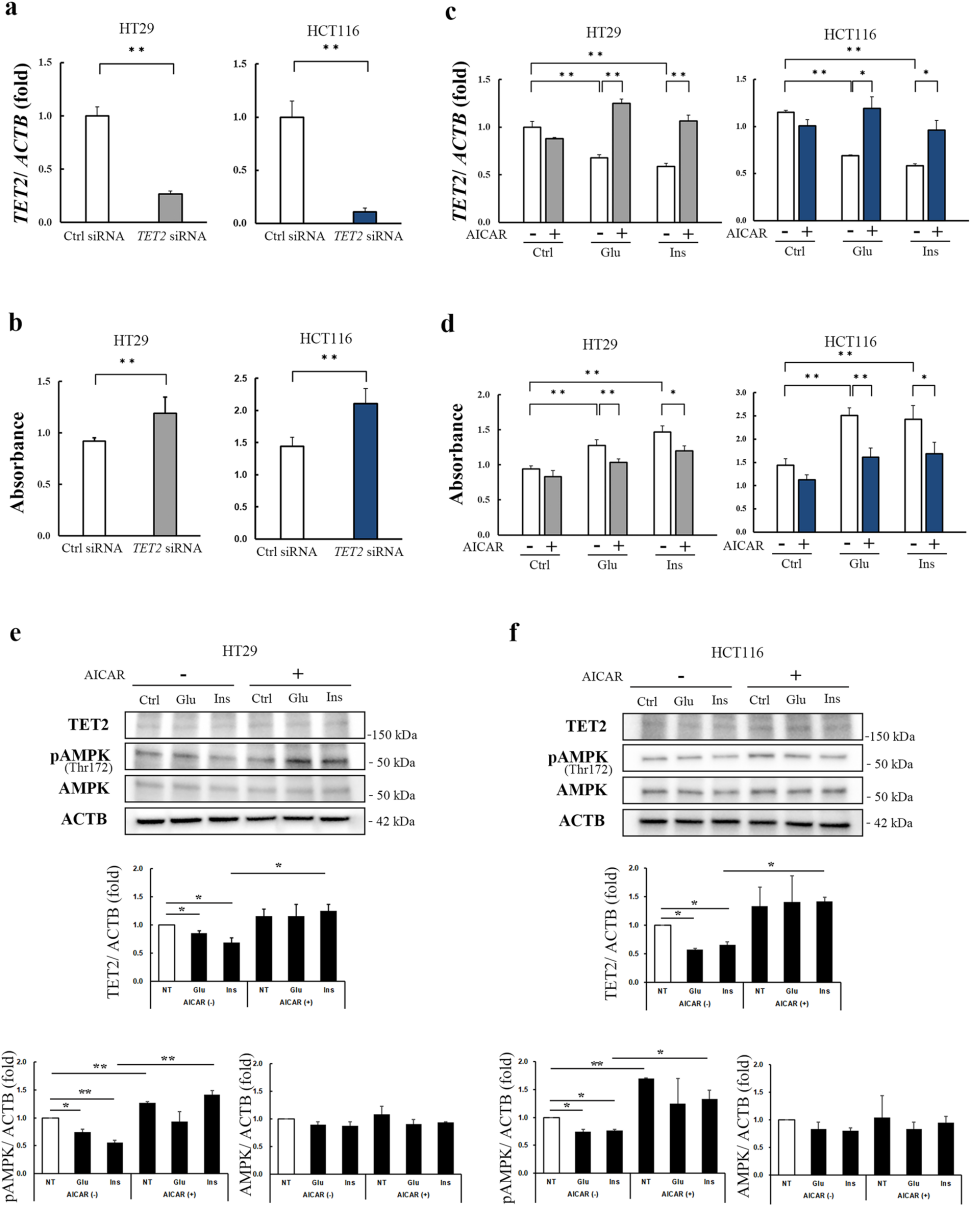
Impact of modulation of *TET2* and AMPK on the proliferation of human colorectal cancer cells. *TET2* siRNA significantly downregulated *TET2* mRNA (*p* < 0.01, **a**) and stimulated the proliferation of both HT-29 and HCT116 cells (*p* < 0.01, **b**). Treatment of both HT-29 and HCT116 cells with AICAR, an AMPK inducer, restored the *TET2* expression that was suppressed by glucose or insulin treatment (**c**) and inhibited the cell proliferation stimulated by glucose or insulin treatment (**d**). Representative western blots show that glucose or insulin treatment reduced TET2 and phosphor-AMPK α expression, which was restored by AICAR treatment in the both HT-29 (**e**) and HCT116 cells (**f**). Densitometry analysis; data are expressed as fold over Ctrl without AICAR treatment (**e**, **f**). *p < 0.05, **p < 0.01. *TET* ten-eleven translocation *ACTB β-actin*, *Ctrl* control, *Glu* glucose, *Ins* insulin, *AICAR* 5-aminoimidazole-4-carboxamide-1-β-D-ribofuranoside, *AMPK* adenosine monophosphate-activated protein kinase.

Using siRNA, a *TET2* knockdown was created (*p* < 0.01, Fig. 7a), which induced proliferation in the CRC cell lines (*p* < 0.01, Fig. 7b). Exposure to 5-aminoimidazole-4-carboxamide-1-β-D-ribofuranoside (AICAR), an analog of AMPK and an inducer of TET2,^22^ restored *TET2* mRNA expression, which was initially reduced by glucose or insulin treatment (Fig. 7c). Furthermore, AICAR treatment inhibited the glucose- or insulin-stimulated cell proliferation in both CRC cell lines (Fig. 7d). A western blot analysis revealed that in both the CRC cell lines, glucose and insulin treatments reduced TET2 and phospho-AMPK α expression, which was restored by AICAR treatment (Fig. 7e, f).

## Discussion

In this study, 5-hmC levels of DNA isolated from FFPE, evaluated using ELISA, and the expression of *TET2* were significantly decreased in Ob-CRC. In CRC cell lines, treatment with glucose- or insulin-stimulated cell proliferation and decreased the expression of TET2. Exposure to AICAR, an AMPK activator, increased glucose- or insulin-suppressed expression of TET2, and inhibited cell proliferation. We demonstrated that the reduction in 5-hmC associated with a decrease in TET2 expression, which reflects the inhibition of demethylation, is involved in the development of obesity-related CRC with insulin resistance and hyperglycemia.

Variations in the epigenetic regulation of gene expression, without changes in DNA sequence, result in various diseases, such as cancer, obesity, neurodegenerative diseases, and psychiatric disorders^24–27^. One of the major epigenetic modifications is DNA methylation, which is the addition of a methyl group at the carbon-5 position of a cytosine base. TET promotes the demethylation process by catalyzing the oxidative conversion from 5-mC to 5-hmC^28^. Nuclear localization of TET2 was lost in a significant portion of the CRC tissues, and nuclear export inhibitors increased the 5-hmC level by regulating TET2 in CRC cells^29^. In this study, the nuclear expression of TET2 was reduced in CRC tissues. There were no differences between the nOb and Ob in global 5-hmC levels of whole genomic DNA; however, 5-hmC and *TET2* mRNA levels were decreased in Ob-CRC tissue. This suggests that the differences in 5-hmC levels in CRC tissues between Ob-CRC and nOb-CRC are due to a tumor-localized effect. Although the immunohistochemistry analysis did not exactly validate these results, we speculate that this discrepancy is due to its low sensitivity as a semi-quantitative technique. We believe that the reduction of 5-hmC associated with decreased *TET2* expression and nuclear localization of TET2 may influence the development of obesity-related CRC.

In addition to aging and environmental factors, obesity is an important factor in the accumulation of DNA damage^30^. We have previously reported that plasma levels of 8-hydroxydeoxyguanosine, which reflect DNA damage caused by reactive oxygen species, are associated with the development of CRA and CRC^31^. 5-hmC is locally deposited at the site of DNA damage by TET, and it plays an important role in promoting DNA repair and maintaining genome integrity^32^. Downregulation of *TET2* may promote the development of obesity-related CRC by inhibiting the protective effect of 5-hmC on DNA damage.

In this study, treatment with glucose or insulin decreased the expression of *TET2*, but treatment with fatty acids did not influence the expression of *TET2*. These results suggest that hyperinsulinemia and hyperglycemia, observed in patients with visceral fat obesity, may decrease the expression of *TET2*. AMPK, which is a key regulator as an energy sensor for maintaining homeostasis, is inactivated in hyperglycemic conditions and activated upon energy stress induced by ATP depletion, such as in hypoglycemia and hypoxia^33,34^. Activated AMPK modulates TET2 stability through phosphorylation of serine 99 on TET2 (TET2S99), while the inactivated AMPK in hyperglycemic cells inhibits the phosphorylation of TET2S99, causing instability of TET2 and decreasing 5-hmC. Treatment with metformin, which activates AMPK, suppresses tumor growth by regulating TET2 in diabetic mice transplanted with malignant melanoma cells (A2058)^22^. The direct effect of insulin on TET2 expression is not known; however, insulin is known to influence the activity of AMPK and glucose^36^. TET2 expression is an independent factor for recurrence and survival of patients with CRC, and the prognostic value of AMPK and TET2 levels in combination was greater than that of the individual markers^37^. In this study, the decrease in the expression of *TET2* because of glucose or insulin treatment in CRC cells was inhibited by AICAR. Therefore, in obesity-related CRC, hyperinsulinemia and hyperglycemia may be involved in the decreased expression of TET2 by an AMPK-mediated mechanism.

Kang et al.^36^ examined the association between the immunohistochemical expression levels of AMPK/TET2 in paraffin-embedded CRC specimens and the prognosis of CRC patients. High expression levels of TET2 were predictive of a favorable prognosis, whereas AMPK was not a significant determinant of patient prognosis. This study did not clarify the effects of 5-hmC levels, obesity, and metabolic factors on the expression of TET2 and AMPK in CRC. Wu et al.^22^, mainly using a malignant melanoma cell line, found that sustained hyperglycemia destabilized TET2 in an AMPK-dependent manner and deregulated 5-hmC. However, they did not study the glucose–AMPK–TET2–5hmC axis in CRC or the effect of insulin on this axis. Our study is the first to show that 5-hmC levels and TET2 expression are reduced in Ob-CRC tissues and that glucose and insulin treatments reduce TET2 expression in CRC cells, partially because of AMPK activity.

5-hmC and TET2 play a significant role in the pathogenesis of insulin resistance in adipose tissues^37^. A TET2 genomic variant (rs9884482) is associated with the levels of fasting plasma insulin^38^. In 3T3-L1 adipocytes, 5-hmC colocalizes with peroxisome proliferator-activated receptor γ (PPARγ) bound to the enhancers. PPARγ is a nuclear receptor associated with energy homeostasis and insulin sensitivity^39^. Sorted from the visceral adipose tissue of healthy humans, PPARγ-positive cells are strongly co-enriched with 5-hmC^40^. Expression of TET2 in adipocytes is downregulated in diet-induced obese mice, and TET2 facilitates PPARγ agonist–mediated gene regulation and insulin sensitization in adipocytes^41^. Moreover, functional knockdown of TET2 in hematopoietic cells increases the expression of IL-1β in the white adipose tissue and induces systemic insulin resistance^23^. Therefore, TET2 plays an active role in the pathogenesis of insulin resistance in adipose tissue. In this study, we observed that TET2 and 5-hmC expression was reduced in patients with CRC with visceral fat obesity, insulin resistance, and hyperglycemia. In addition to its role in the adipose tissue, TET2 may be involved in obesity-related CRC development. Regulating TET2 might lead to the amelioration of obesity-related pathological conditions.

Epigenetic gene regulation is a reversible change that can have therapeutic applications. Azacitidine, a DNA methyltransferase inhibitor, is mainly used in the treatment of myelodysplastic syndrome^42^. Vorinostat, a histone deacetylase inhibitor, is used for treating cutaneous T-cell lymphoma^43^. Metformin, which activates AMPK, has a TET2-dependent anti-tumor effect in diabetic nude mice transplanted with malignant melanoma cells (A2058)^22^. In this study, activation of AMPK by AICAR inhibited insulin- or glucose-stimulated cell proliferation and decreased the expression of TET2. These findings imply that TET2 stabilization appears to be a potential therapeutic target for obesity-related CRC. This could lead to personalized medicines targeting the specific metabolic alterations in individual CRC patients.

There are some limitations to this study. Our observations are from a small number of patients, and it is necessary to verify the results in a large number of patients at various stages of CRC. The mechanism underlying why only TET2, among the TET family members, is susceptible to obesity and metabolic alterations in CRC remains unclear. It is crucial to elucidate the mechanism underlying this selectivity in future studies. The TET2-mediated anti-tumor effects of AICAR on CRC in vivo and the clinical significance of the results of this study require further evaluation.

In conclusion, we showed for the first time that the expression of 5-hmC and TET2 was downregulated in CRC with hyperglycemia and insulin resistance associated with visceral fat obesity in this study. Glucose or insulin altered AMPK-mediated TET2 expression and influenced the proliferation of CRC cells. TET2-mediated epigenetic modifications, influenced by systemic metabolic alterations, are proposed as a novel developmental mechanism in obesity-related CRC, and therefore, may be a promising therapeutic target.

## Methods

### Study subjects

From April 2017 to March 2019, we retrospectively screened 81 CRC patients, who underwent colonoscopy and surgical resection at Yamagata University Hospital. VFA was measured in each subject using preoperative computed tomography images at the umbilical level (fatAnalyse on EV Insite R, Public and social system Solution Provider Corporation, Tokyo, Japan). We defined obesity as a VFA greater than 100 cm^2^. On the basis of the 8th Union for International Cancer Control (UICC), we randomly selected seven Ob-CRC among the 81 CRC patients, excluding stage IV CRC patients^44^. Again on the basis of the 8th UICC, we selected seven nOb-CRC and ensured that they matched the seven Ob-CRC patients in terms of age, sex, histological type, location, and CRC stage. None of the eligible patients had received preoperative anticancer therapy. This study was conducted in accordance with the Declaration of Helsinki and approved by the Ethical Review Committee of Yamagata University Faculty of Medicine (Approval Number: 2018-440). Informed consent was waived by the Ethical Review Committee of Yamagata University Faculty of Medicine due to the retrospective nature of this study.

### Preoperative clinical data acquisition

Clinical information about height, weight, smoking, alcohol consumption, and history of medications was obtained from medical records. Current smoking habit was defined as smoking at least one cigarette daily within the past 12 months. Alcohol consumption was defined as alcohol intake of > 25 g/day. Trained nurses obtained anthropometric measurements of the participants, including their height and weight. BMI was calculated as weight in kilograms divided by height in meters^2^. Fasting blood samples were collected from all subjects after an overnight fast, and samples were immediately separated into serum and plasma. These samples were analyzed for serum concentrations of TC, LDL, HDL, TG, HbA1c, FPG, and FPI. The index of insulin sensitivity was evaluated using the HOMA-IR and calculated as follows: HOMA-IR = FPI (µIU/mL) × FPG (mmol/L)/22.5. Using hematoxylin and eosin staining, two pathologists at Yamagata University Hospital diagnosed all surgical specimens with colorectal adenocarcinoma.

### Immunohistochemistry

The 3 µm FFPE sections were deparaffinized and boiled in 10 mM citric buffer (pH 6.0) for antigen retrieval. After blocking with BLOXALL blocking buffer (Vector Laboratories, UK) and 2.5% normal horse serum (Vector Laboratories, UK), the tissues were incubated with anti-TET2 antibody (1:200, ab94580, Abcam, Cambridge, UK) or anti-5-hmC antibody (1:200, #39769, ACTIVE MOTIF, Japan) at 4 °C overnight. The tissues were treated with ImmPRESS universal antibody polymer reagent (made in a horse, Vector Laboratories, UK), followed by diaminobenzidine staining using ImmPACT DAB EqV peroxidase substrate (Vector Laboratories, UK). Images were obtained using light microscopy (BX53, Olympus, Tokyo, Japan). The high- and low-power field images were captured at 400× and 100× magnification, respectively.

Immunohistochemistry results were evaluated using a semi-quantitative approach to assign an H-score to tissue samples^24^. The nuclei staining intensity of 0, 1+ , 2+ , or 3+ was determined for each cell in a fixed field corresponding to the presence of negative, weak, intermediate, and strong staining, respectively. Subsequently, the amount of cells at each staining intensity level was calculated in percentages, and finally, H-scores (0–300) were assigned using the following formula: H-score = 1 × (percentages of 1 + cells) + 2 × (percentages of 2 + cells) + 3 × (percentages of 3 + cells). The H-scores for each case were calculated as the mean scores of the three individual sections.

### Cell culture

Human CRC cell lines (HT29, ATCC-HTB-38, VA, USA; HCT116, ATCC-CCL247, VA, USA) were maintained in McCoy’s 5A medium (Thermo Fisher Scientific, USA) supplemented with 10% fetal bovine serum (Thermo Fisher Scientific, USA) and 1% penicillin/streptomycin (Thermo Fisher Scientific, USA) at 37 °C with 5% CO_2_ in a humidified incubator (MCO-230AICUVH-PJ, Panasonic, Japan).

### Cell proliferation assay

HT29 and HCT116 cells were cultured in 96-well plates (2 × 10^4^ cells/well) and maintained for 24 h. Then, the cell culture medium was replaced with McCoy’s 5A medium without serum and incubated at 37 °C with 5% CO_2_ in a humidified atmosphere for 12 h. Subsequently, the cells were treated with 50 mM glucose (Wako, Osaka, Japan), 100 nM recombinant human insulin (Novo Nordisk Pharma, Tokyo, Japan), 250 µM OA (Sigma-Aldrich, Germany), 250 µM PA (Sigma-Aldrich, Germany)^45^, 100 µM AICAR (AdipoGen, CA, USA), and phosphate-buffered saline as control (Ctrl) for 6 h, or siRNA for 72 h. After treatment, 10 µL of Premix WST-1 solution (TaKaRa, Japan) was added to each well and incubated for 6 h at 37 °C. After incubation, the absorbance was measured at 450 nm using a microplate reader (Benchmark Plus Microplate Reader, Bio-Rad, USA), at a wavelength of 690 nm.

### 5-hydroxymethylcytosine quantification

Genomic DNA was purified from the blood samples using a QIAamp DNA Blood Mini Kit (QIAGEN, Germany). CRC tissue and NC tissues were separately dissected from 10 μm FFPE specimens. Subsequently, DNA was extracted from the isolated FFPE specimens using the GeneRead DNA FFPE kit (QIAGEN, Germany). The extracted DNA was quantified using a Qubit dsDNA HS (high sensitivity) assay kit (Thermo Fisher Scientific, USA). Using 100 ng DNA from each sample, 5-hmC levels were determined in duplicate using a MethylFlash global hydroxymethylation ELISA easy kit (EpiGentek, USA), in accordance with the manufacturer's instructions. The values presented here are the mean of the 5-hmC levels from the duplicate samples. The intraclass correlation coefficient between the duplicates was 0.82.

### siRNA transfection

Silencer pre-designed *TET2* siRNA (AM16708, ID:126964, Ambion) and negative control No.1 siRNA (AM4611, Ambion) were purchased from Thermo Fisher Scientific. *TET2* siRNA and negative control siRNA (10 µM) were transfected into HT-29 and HCT116 cells using Lipofectamine RNAiMAX transfection reagent (Invitrogen, USA), according to the manufacturer’s protocol. After 72 h of incubation, the cells were used for the subsequent experiments.

### RNA extraction, reverse transcription, and quantitative real-time polymerase chain reaction

Total RNA was extracted from FFPE tissues or cultured cells using the RNeasy FFPE kit (QIAGEN, Germany) or RNeasy Plus Mini Kit (QIAGEN, Germany), according to the manufacturer's protocol. One microgram of RNA was reverse transcribed into cDNA using the iScript cDNA synthesis kit (Bio-Rad, USA), and RT-qPCR was performed on the 7500 Fast Real-Time PCR System (Applied Biosystems, USA) using PowerUp SYBR Green Master Mix (Applied Biosystems, USA). The primers used for RT-qPCR are listed in Supplementary Table 2. The absolute amount of target genes was calculated based on the standard curve generated from the templates and the gene expression was normalized based on the expression of the housekeeping gene, *β-actin* (*ACTB*).

### Western blot analysis

Whole cell protein lysates were prepared using radioimmunoprecipitation lysis and extraction buffer, containing halt protease and phosphatase inhibitor cocktail, according to the manufacturer’s instruction (Thermo Fisher Scientific, USA). Protein concentrations were measured using the Pierce BCA protein assay kit (Thermo Fisher Scientific, USA). Twenty micrograms of protein samples was electrophoresed on a 4–15% precast TGX polyacrylamide gel (Bio-Rad, USA) and transferred onto PVDF membranes using Trans-Blot Turbo Transfer System (Bio-Rad, USA). After blocking with the Every blot blocking buffer (Bio-Rad, USA), membranes were incubated with the primary antibodies against TET2 (1:2000, NBP2-32104, Novus Biologicals, USA), phosph-Thr172 AMPK α (1:1000, #2535, Cell Signaling, USA), AMPK α (1:1000, #2532, Cell Signaling, USA), and ACTB (1:10000, GTX109639, GeneTex, Taiwan) overnight at 4 °C. The membranes were incubated with anti-rabbit goat IgG-peroxidase conjugate antibody (7074P2, Cell Signaling, USA) for 1 h. The immunoreactive proteins on the membranes were visualized using the Clarity western ECL substrate (Bio-Rad, USA) and ChemiDoc XRS + system (Bio-Rad, USA). Densitometric analysis was performed using Image J 1.53t software.

### Statistical analysis

Data are expressed as medians (interquartile range) or numbers (percentage). For the comparisons between two groups, continuous variables or categorical variables were analyzed using the Wilcoxon rank sum test or χ2 test, respectively. All statistical analyses were performed using JMP v14.2.0 (SAS Institute Inc., NC, USA). A *p*-value less than 0.05 (*p* < 0.05) was considered statistically significant.

## Supplementary Information

Supplementary Information.

## Data Availability

The datasets used and/or analyzed during the current study are available from the corresponding author on reasonable request.
