# Supplementary material for: Modulation of AMPK/ TET2/ 5-hmC axis in response to metabolic alterations as a novel pathway for obesity-related colorectal cancer development
Source: Sci Rep. 2023 Feb 17;13:2858. doi: 10.1038/s41598-023-29958-2 (PMC9938119; doi:10.1038/s41598-023-29958-2)

Modulation of AMPK/ TET2/ 5-hmC axis in response to metabolic alterations as a novel pathway for obesity-related colorectal cancer development

Takashi Kon[1], Yu Sasaki[1], Yasuhiko Abe[2], Yusuke Onozato[1], Makoto Yagi[2], Naoko Mizumoto[1], Takayuki Sakai[1], Matsuki Umehara[1], Minamin Ito[1], Shuhei Nakamura[1], Hiroki Goto[1], Yoshiyuki Ueno[1]

[1]Department of Gastroenterology, Faculty of Medicine, Yamagata University, 2-2-2 Iida-Nishi, Yamagata 990-9585, Japan

[2]Division of Endoscopy, Yamagata University Hospital, 2-2-2 Iida-Nishi, Yamagata 990-9585, Japan

Corresponding author

Yu Sasaki, MD, PhD

Faculty of Medicine, Department of Gastroenterology, Yamagata University

2-2-2 Iida-Nishi, Yamagata 990-9585, Japan

Tel: +81-23-628-5309 Fax: +81-23-628-5311

E-mail: y-sasaki@med.id.yamagata-u.ac.jp

Supplementary information accompanying this paper

Supplementary Table 1. Comparison of selected characteristics between obese patients with CRC and non-obese patients with CRC

Characteristic	Ob-CRC (*n* = 7)	nOb-CRC (*n* = 7)	*p*-value
Age (year)	77 (65–80)	77 (71–83)	NS (matched)
Male, *n* (%)	4 (57)	3 (43)	NS (matched)
Current smoking, *n* (%)	1 (14)	1 (14)	NS
Alcohol consumption, *n* (%)	2 (29)	1 (14)	NS
VFA (cm^2)	185.6 (152.1–237.0)	31.6 (27.1–96.6)	< 0.01
BMI (kg/m^2) [a]	28.7 (27.7–29.2)	20.9 (19.1–22.5)	< 0.01
Diabetes, *n* (%)	4 (57)	1 (14)	NS
Dyslipidemia, *n* (%)	4 (57)	2 (29)	NS
FPG (mmol/L)	6.0 (5.4–6.2)	4.9 (4.8–5.3)	< 0.01
HbA1c (%)	6.3 (5.8–7.2)	5.8 (5.5–6.6)	NS
FPI (μU/mL)	7.6 (5.4–12.1)	4.6 (3.1–4.9)	< 0.05
HOMA-IR [b]	2.0 (1.5–3.3)	1.1 (0.7–1.2)	< 0.05
TG (mmol/L)	1.0 (0.8–2.5)	1.7 (1.4–2.0)	NS
TC (mmol/L)	4.7 (3.5–5.0)	4.8 (3.7–6.2)	NS
HDL (mmol/L)	1.2 (1.0–1.4)	1.22 (1.0–1.4)	NS
LDL (mmol/L)	3.0 (2.1–3.2)	3.0 (2.3–3.9)	NS
CRC stages [c], *n* (%)			NS (matched)
I	2 (29)	2 (29)	
II	4 (57)	3 (43)	
III	1 (14)	2 (29)	
IV	0 (0)	0 (0)	
Tumor location [d]			NS (matched)
Right side, *n* (%)	3 (43)	3 (43)	
Left side, *n* (%)	4 (57)	4 (57)	

Values are expressed as medians (interquartile range) or numbers (percentage). *p*-values

were obtained using Wilcoxon rank-sum test or the χ^2 test. [a] BMI was calculated as weight in kilograms divided by height in meters. [b] HOMA-IR = FPI (μIU/mL) × FPG (mmol/L)/22.5. [c] Classification of CRC patients based on the 8th UICC staging system. [d] Right-sided colon and left-sided colon were defined, as colorectal cancer was located in the proximal to transverse colon and descending colon to rectum, respectively.

Ob-CRC, obese patients with colorectal cancer; nOb-CRC, non-obese patients with colorectal cancer; VFA, visceral fat area; BMI, body mass index; FPI, fasting plasma insulin; HOMA-IR, homeostasis model assessment of insulin resistance; TG, triglyceride; TC, total cholesterol; HDL, high-density lipoprotein; LDL, low-density lipoprotein; NS, not significant; UICC, Union for International Cancer Control.

Supplementary Table 2. Primer sequences used for RT-qPCR

Targets	Primer sequences
**Human *ACTB***	Forward: 5′-TTCCTTCCTGGGCATGGAGTCC-3′
	Reverse: 5′-TGGCGTACAGGTCTTTGCGG-3′
**Human *TET1***	Forward: 5′-CAGAACCTAAACCACCCGTG-3′
	Reverse: 5′-TGCTTCGTAGCGCCATTGTAA-3′
**Human *TET2***	Forward: 5′-GGCTACAAAGCTCCAGAATGG-3′
	Reverse: 5′-AAGAGTGCCACTTGGTGTCTC-3′
**Human *TET3***	Forward: 5′-TCCAGCAACTCCTAGAACTGAG-3′
	Reverse: 5′-AGGCCGCTTGAATACTGACTG-3′

RT-qPCR, reverse transcription-quantitative polymerase chain reaction; *ACTB*, *β-actin*; *TET*, *ten-eleven translocation*.

Supplementary Figure 1. Full-length Western blot images used in Figure 7

Full-length blot images of TET2 (a), phospho-AMPK (b), AMPK (c), and ACTB (d) in Figure 7e. Full-length blot images of TET2 (e), phospho-AMPK (f), AMPK (g), and ACTB (h) in Figure 7f. TET, ten-eleven translocation; AMPK, adenosine monophosphate-activated protein kinase; ACTB, β-actin.

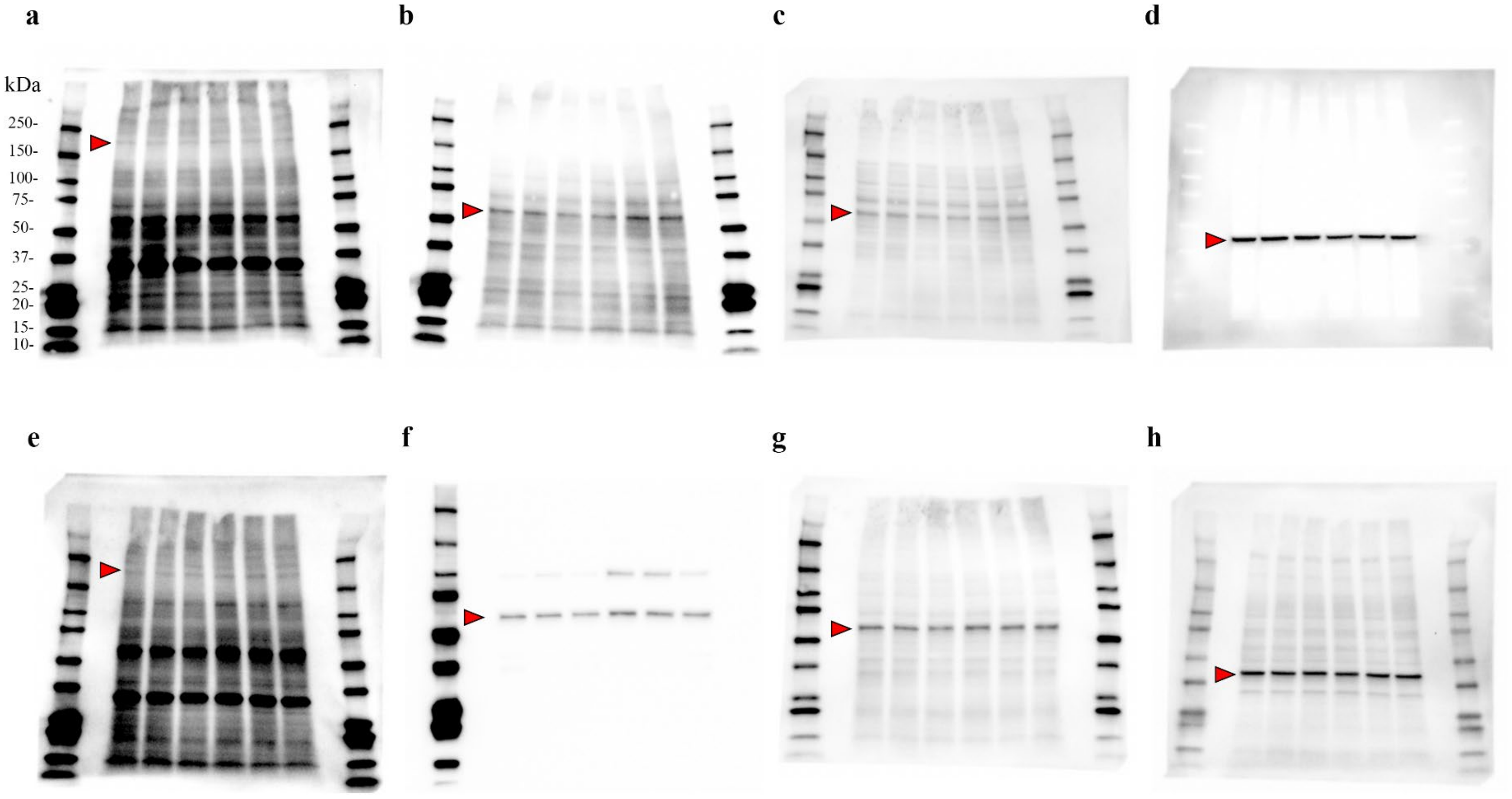

Supplement: Supplementary file 1 — Supplementary Information. [file 41598_2023_29958_MOESM1_ESM.pdf]
